# Option B+ for prevention of vertical HIV transmission has no influence on adverse birth outcomes in a cross-sectional cohort in Western Uganda

**DOI:** 10.1186/s12884-017-1263-2

**Published:** 2017-03-07

**Authors:** Eva M. Rempis, Alexandra Schnack, Sarah Decker, Vera Braun, John Rubaihayo, Nazarius Mbona Tumwesigye, Priscilla Busingye, Gundel Harms, Stefanie Theuring

**Affiliations:** 1 Institute of Tropical Medicine and International Health, Charité- University Medicine, Augustenburger Platz 1, Berlin, 13353 Germany; 2 Department of Public Health, Mountains of the Moon University, Fort Portal, Kabarole Uganda; 3 School of Public Health, College of Health Sciences, Makerere University, Kampala, Uganda; 4 The Holy Family Virika Hospital, Fort Portal, Kabarole Uganda

**Keywords:** Human immunodeficiency virus type 1 (HIV-1), Antiretroviral therapy (ART), Prevention of mother-to-child-transmission (PMTCT), Option B+, Adverse pregnancy (birth) outcomes, Stillbirth, Preterm delivery, Small for gestational age, Uganda

## Abstract

**Background:**

While most Sub-Saharan African countries are now implementing the WHO-recommended Option B+ protocol for prevention of vertical HIV transmission, there is a lack of knowledge regarding the influence of Option B+ exposure on adverse birth outcomes (ABOs). Against this background, we assessed ABOs among delivering women in Western Uganda.

**Methods:**

A cross-sectional, observational study was performed within a cohort of 412 mother-newborn-pairs in Virika Hospital, Fort Portal in 2013. The occurrence of stillbirth, pre-term delivery, and small size for gestational age (SGA) was analysed, looking for influencing factors related to HIV-status, antiretroviral drug exposure and duration, and other sociodemographic and clinical parameters.

**Results:**

Among 302 HIV-negative and 110 HIV-positive women, ABOs occurred in 40.5%, with stillbirth in 6.3%, pre-term delivery in 28.6%, and SGA in 12.2% of deliveries. For Option B+ intake (*n* = 59), no significant association was found with stillbirth (OR 0.48, *p* = 0.55), pre-term delivery (OR 0.97, *p* = 0.92) and SGA (OR 1.5, *p* = 0.3) compared to seronegative women. Women enrolled on antiretroviral therapy (ART) before conception (*n* = 38) had no different risk for ABOs than women on Option B+ or HIV-negative women. Identified risk factors for stillbirth included lack of formal education, poor socio-economic status, long travel distance, hypertension and anaemia. Pre-term delivery risk was increased with poor socio-economic status, primiparity, Malaria and anaemia. The occurrence of SGA was influenced by older age and Malaria.

**Conclusion:**

In our study, women on Option B+ showed no difference in ABOs compared to HIV-negative women and to women on ART. We identified several non-HIV/ART-related influencing factors, suggesting an urgent need for improving early risk assessment mechanisms in antenatal care through better screening and triage systems. Our results are encouraging with regard to continued universal scale-up of Option B+ and ART programmes.

## Background

Maternal HIV infection can increase the occurrence of adverse birth outcomes (ABOs), such as pre- term delivery (PTD), stillbirth (SB), or newborns too small for gestational age (SGA) [1–3]. Not only HIV infection itself, but also HIV- associated conditions like lower maternal body weight, anaemia, sexually transmitted infections, Malaria, or Tuberculosis [4–7] are associated with higher rates of ABO. Prophylactic antiretroviral (ARV) drug regimens during pregnancy are not only an instrument for prevention of mother-to-child-transmission of HIV (PMTCT), but can also improve the mother’s health and positively influence conditions associated with ABO occurrence [8]. Since 2012, the WHO has recommended “Option B+” for PMTCT [9], which stands for initiation of life-long antiretroviral therapy (ART) among all HIV- positive pregnant women. This approach has since then been further strengthened by the trend to lower thresholds for ART initiation, particularly by the recent WHO release of “treat all”- recommendations, pursuing ART for all HIV-positive individuals including pregnant women. Consequently, the vast majority of PMTCT clients in the near future will be exposed to lifelong ART [8].

However, ARV drugs themselves may also negatively influence the occurrence of ABOs, and thus increase perinatal morbidity and mortality. Research has shown higher rates for SB [10, 11], PTD [12–15] and SGA [11, 16, 17] for infants of women who take ARVs for treatment or prevention. SGA and PTD do not only have an immediate influence on perinatal mortality, but have repercussions on the under-1-year and even under-five year infant morbidity and mortality [18, 19]. While it seems that ARV regimens containing protease inhibitors (PI) have a higher influence on the occurrence of ABOs [17, 20–23], possible relations were also discovered for Nucleotide/Nucleoside reverse transcriptase inhibiting (N(t)RTI) and non- NRTI (NNRTI) drugs now recommended as Option B+ regimen [10, 12–14]. There also seems to be an association between ABOs and the duration of drug intake and time of drug initiation, with pre-conception drug intake linked with higher odds for ABOs than initiation during pregnancy [10, 24, 25].

HIV serostatus and different ARV drug regimens for HIV seropositive women may thus both negatively influence birth outcome. Increasing numbers of women and infants will benefit from the therapeutic and preventive effects of ARV intake during pregnancy. In this light, more information about possible adverse effects of ARV exposure is required. Since ABOs have substantial impact on the overall morbidity and mortality of infants, we aimed to understand their association with different types and duration of ARV exposure (ART or Option B+) in pregnancy.

In Uganda, an estimated 1.6 million people are living with HIV/AIDS, of which around 170 thousand are children [26]. While the nation-wide prevalence of HIV in pregnant women is 6.1% [27], the prevalence of 13.4% in Kabarole district, Western Uganda, is among the highest in the country [28]. The total fertility rate of Ugandan women is 6.0 children. The neonatal mortality rate is estimated at 23/1000, and the SB rate at 25/1000 births. Low birth weight incidence was 14% in 2011 [29]. As of 2011, 57.4% of women delivered in a health facility [29]. The implementation of Option B+ in Uganda for PMTCT started at the end of 2012. Our study aimed at assessing ABOs among women and their newborns in Fort Portal, Uganda, under consideration of ARV exposure during pregnancy.

## Methods

We conducted a cross-sectional study to investigate the occurrence of birth outcomes like SB, PTD and SGA among a cohort of delivering women in Fort Portal, Uganda. The primary objective was to assess possible influence of HIV serostatus and ARV exposure duration and type (ART or Option B+). Furthermore, we assessed associations of non-HIV related factors to the occurrence of ABO.

### Study site

Virika Hospital, a private catholic hospital representing one of two referral hospitals of Kabarole District, offers standard antenatal, delivery and HIV care and contains a 32-bed obstetric department. Around 10% of delivering women are HIV-infected in this setting. For women identified HIV-positive during pregnancy, Option B+ for PMTCT is applied since September 2012. For assumed seronegative delivering women, HIV status is reassessed peripartumly as a routine procedure. In accordance with the Ugandan PMTCT protocol, all tested women receive pre- and post-test counselling, and those who test positive are counselled and immediately enrolled in Option B+ drug regimen. All women receive adequate standard obstetrical care.

### Data collection

From February until December 2013, women who came to Virika Hospital for delivery were recruited into the study. Eligibility criteria included age 18 years or older, singleton pregnancy, informed written consent, and known HIV serostatus (as confirmed in routine peripartum testing). Upon recruitment, we obtained socio-demographic, obstetric, and medical data by using an interviewer-administered, structured questionnaire based on women’s self-report. Medical data involved history of Malaria infection and HIV-related data, which included prophylactic and therapeutic measures during the current and former pregnancies. Women were examined clinically and obstetrically, and a venous blood sample was taken. Malaria status at delivery was determined by microscopy and a rapid test device. Haemoglobin (HB) level was determined using photometer technique. The newborn’ s birth outcome and weight were documented. Gestational age in weeks (GW) of the newborns was assessed using the Finnstroem scoring system [30].

The outcome variables were defined as follows: SB was determined as a newborn above 28 GW, delivered with an APGAR score of 0 in the first and after five minutes. PTD was specified as a newborn with an equivalent score of 37 GW or less according to the Finnstroem score assessment. We defined infants to be SGA if they had a birth weight below the 10^th^ percentile of a foetal growth chart. Since to date no growth chart is available for Uganda, we used a chart developed for Tanzania [31]. Due to cultural reasons, stillborn infants were often taken away by the family before they could be assessed and weighed. Some SB babies were therefore missing in the PTD and SGA analysis, but all were included in the SB analysis.

The explanatory variable “HIV-negative” was defined as having had a negative bedside peripartum test; “ART pre-conception” (ARTpc) was specified as having commenced a highly active ARV treatment (Tenofovir, Lamivudine and Efavirenz as recommended first-line regimen) before conception of the current pregnancy. The definition of “Option B+” was the intake of the ARV combination Tenofovir, Lamivudine and Efavirenz, initiated during the current pregnancy. Date of treatment/PMTCT start was extracted from the ANC card, or self- reported if ANC card was missing.

Since it was shown that a protective effect of PMTCT due to viral load reduction requires a minimum of 90 days of ARV intake [32–34], we used this as cut-off point for analysing influence of ARV intake duration before delivery. A second cut-off point was set at 14 GW, as ARV exposure early in pregnancy is particularly suspected to influence ABO occurrence [10, 12, 24, 35, 36].

Six women referred from external facilities were still enrolled on the “Option A” regimen (Zidovudine after GW 14 and Co-trimoxazole) at the time of delivery. For two women, the PMTCT regimen was not specified. These were included in analyses concerning serostatus and non- HIV related explanatory factors for ABO, but excluded from ARV-related analysis. All women on Option A were changed on Option B+ regimen after delivery and referred to chronic HIV care. Only five HIV-positive women were not on ARVs, with two of them not being aware of their seropositivity. They were included in descriptive baseline analysis, but excluded from analysis of HIV/ARV related parameters.

As non-HIV related explanatory variables for ABO, haemoglobin level of 11.5 g/dl and less was defined as anaemia, using the WHO definition of 11 g/dl, adding 0.5 g/dl to adjust for the study site’s altitude of 1550 m [37]. The occurrence and gestational week of Malaria in pregnancy (MIP) were either extracted from the ANC card or self- reported by the women. Where the last normal menstrual period date was available, MIP was classified as having occurred in the first trimester, or after start of the second trimester. Women whose laboratory examination confirmed Malaria infection upon delivery were included into the variable “Malaria within two weeks prior delivery”. The variable “any obstetric risk history” consisted of self-reported obstetric history of abortion, stillbirth, preterm delivery and preterm labour and clinical findings during this current pregnancy, including hypertension, pre-eclampsia, or sexually transmitted diseases.

Socio-economic status (SES) was classified by using a scale of self-reported availability of resources in the woman’s household as proxies, such as tap water, electricity, refrigerator, motorbike/car, cattle, cupboard, and television. Lowest SES was defined as having none of the proxy assets available in the household. We also assessed whether the women benefitted from transport and delivery cost coverage through support organisations (e.g., Baylor Uganda).

### Data analysis

Data was entered into Excel (Microsoft) data sheets and checked for consistency. Data analysis was carried out using IBM SPSS Statistics, version 22.0. We performed descriptive analysis of the sociodemographic, economic and clinical background of clients and tested for differences between HIV-positive and negative women. Clinical outcomes of newborns were described and equally tested for differences according to HIV-exposure. For univariate analysis, Mann- Whitney or Kruskal-Wallis test was used for continuous data, and Pearson’s Chi Square and Fisher’s exact test (as appropriate) for categorical data. We looked for factors influencing the outcome variables SB, PTD, SGA calculating odds ratios (OR). Explanatory variables which were significant in univariate analysis were included into multivariate logistic regression to calculate adjusted odds ratios (AOR). A significant *p*-value ≤0.05 and a confidence interval of 95% was used for all analyses.

## Results

### Socio-demographic characteristics

From 912 deliveries in the recruitment period, 412 mother-newborn-pairs fulfilled eligibility criteria for inclusion into the study; 445 (48.8%) had to be excluded from the cohort because of missing peripartum serostatus determination, 55 due to other non- eligibility criteria.

Within our cohort 302 women were confirmed seronegative, and 110 were confirmed seropositive. Among HIV-positive women, 38 (34.5%) had already been on ART before conception, and 59 (53.6%) were enrolled in the Option B+ PMTCT programme (five women untreated, eight women on other PMTCT regimens). Of the women taking ARTpc or Option B+, 81 (84.4%) commenced drug intake a minimum of 90 days before delivery, while 15 (15.6%) received Option B+ for less than 90 days (one missing data on intake duration). The median intake of Option B+ was 131 days prior to delivery. The five untreated seropositive participants showed no clinical signs of advanced disease, anaemia was documented in two cases.

Basic sociodemographic data are shown in Table 1. Participants were most frequently from Kabarole district and Batooro ethnicity. Travel distance to Virika Hospital was on average 60 min. While 168 (41.1%) women were referred for deliveries from lower level healthcare services, free of charge transport and delivery cost coverage was provided for 72 (18.7%). Almost all women (97.5%) had presented at least once to ANC services, with an average of 4 visits during the pregnancy. The majority (90.4%) of women had received Malaria prophylaxis.

**Table 1:** Sociodemographic and clinical baseline data, difference by HIV status

Variables	Overall	HIV negative	HIV positive	OR^a^	CI 95%	*P* value^b^
N total (%)	412 (100)	302 (73.3)	110 (26.7)			
Age (years)^c^	25 (18–42)	25 (18–42)	26 (18–42)			0.052^d^
No. of persons in household^c^	4 (1–22)	4 (1–22)	3.5 (1–10)			0.08^d^
Single/widowed/divorced ^e^	67/410 (16.3)	40 (13.3)	27 (24.8)	2.15	1.24–3.72	*0.005*
Education: primary and less^e^	242/405 (59.8)	168 (56.4)	74 (69.2)	1.74	1.09–2.78	*0.021*
Income generating activity^e^	101/394 (25.6)	69 (24.0)	32 (30.2)	1.37	0.84–2.25	0.21
Socioeconomic status: lowest category^e^	92/411 (22.4)	74 (24.6)	18 (16.4)	0.6	0.34–1.06	0.08
Travel distance to hospital ≥90 min^e^	74/374 (19.8)	61 (21.8)	13 (13.8)	0.58	0.3–1.11	0.094
Referral from other health facilityl^e^	168/409 (41.1)	123 (41.1)	45 (40.9)	0.99	0.64–1.55	0.97
Cost coverage grant for transport/delivery^e^	103/397 (25.9)	60 (20.3)	43 (42.2)	2.9	1.76–4.63	*<0.001*
Primiparity^e^	131/409 (32.4)	107 (35.5)	24 (21.8)	0.51	0.3–0.84	*0.008*
Grand multiparity (≥5 deliveries)^e^	48/411 (11.7)	35 (11.6)	13 (11.8)	1.02	0.52–2.01	0.96
Any obstetric risk history^e^	122/401 (30.4)	71 (24.1)	51 (48.1)	2.93	1.84–4.66	*<0.001*
Hypertension^e^	10/412 (2.4)	8 (2.6)	2 (1.8)	0.68	0.14–3.26	0.63
MIP reported^e^	110/402 (27.4)	84 (28.3)	26 (24.8)	0.84	0.5–1.39	0.49
Anaemia ≤11.5 mg/dl^e^	117/373 (31.4)	84 (29.9)	33 (35.9)	1.31	0.8–2.2	0.28
ANC attendance: yes^e^	387/397 (97.5)	286 (97.6)	101 (97.1)	0.82	0.21–3.25	0.78
No. of ANC visits^c^	4 (0–9)	4 (0–9)	4 (0–9)			0.16^d^

^a^All dichotomous variables consist of the respective attribute compared to the converse attribute and were cross tabulated against the women’s serostatus. The results of the converse attribute is not displayed

^b^Bivariate, Pearson’s X2 asymptotic two-sided *p*-value, if not indicated otherwise. *P*-values in italics indicate statistically significant differences between the groups

^c^median (range)

^d^Mann–Whitney-U-Test

^e^n/total n with available data (%)

HIV-infected women were significantly less often in a partner relationship compared to non-infected women (*p* = 0.005). The risk of HIV infection was negatively correlated with school education on a significant level (*p* = 0.021), and increased with parity (*p* = 0.008), and, by trend, with age (*p* = 0.052).

### Obstetric and newborn data

Of the 412 women, 32.4% were primiparae, and 11.7% grand multiparae (five or more deliveries). According to their clinical history, 30.4% of women had an obstetric risk or pathologies within the current pregnancy. HIV-positive women showed increased odds for having an obstetric risk history, especially for previous SB (OR 2.5, CI 1.09–5.77, *p* = 0.027) or preterm labour (OR 5.66 CI 1.02–31.34, *p* = 0.046). 27.4% reported MIP, of which 15.8% occurred within the 3^rd^ trimester, and 6.1% within the last 2 weeks prior to delivery. A positive peripartum bedside Malaria test was found in 13 (3.4%) cases. Average haemoglobin level was 12.23 g/dl (SD 1.98), anaemia was found in 117 (31.4%) of women at delivery.

In our study cohort, 209 male and 201 female singletons were delivered (sex not reported in two cases). Birth weight was significantly lower for female infants (*p*= 0.01). 157 newborns were delivered by caesarean section (38.6%), four (1%) by obstetric operative methods and 246 (60.4%) as spontaneous vaginal delivery (5 missing data). Basic infant data differentiated for maternal HIV serostatus is shown in Table 2.

**Table 2:** Basic Infant data

Variables	Overalll^a^	No HIV exposure N (%)	HIV exposure N (%)	OR	CI 95%	*P* value^b^
N total	412	302	110			
Gestational week (Finnstroem score)^c^	38 (28–42)	39 (28–42)	38 (30–42)			0.5^d^
Birth weight^c^	3095 (500–4500)	3100 (500–4500)	3040 (1200–4500)			0.821^d^
APGAR score^c^	10 (0–10)	10 (0–10)	10 (0–10)			0.34^d^
Newborn sex						
Male	209/410 (51.0)	156 (52.0)	53 (48.2)	1		
Female	201/410 (49.0)	144 (48.0)	57 (51.8)	1.17	0.75–1.8	0.49
Stillbirth	26/412 (6.3)	20 (6.6)	6 (5.5)	0.81	0.32–2.08	0.67
Preterm delivery	116/410 (28.3)	85 (28.2)	31 (28.4)	1.01	0.62–1.64	0.97
Small for gestational age	47/399 (11.8)	33 (11.3)	14 (13.1)	1.18	0.61–2.31	0.63
Any adverse birth outcome	165/409 (40.3)	119 (39.7)	46 (42.2)	1.11	0.71–1.73	0.64

^a^All data n/total n with available respective data (%), if not indicated otherwise

^b^Bivariate, Pearson’s X^2^ asymptotic two- sided *p*- value

^c^Median (range)

^d^Mann–Whitney-U-Tes

### Adverse birth outcomes

ABOs were observed in 165 (40.3%) of women (*n*= 409, 3 missing data for SGA), with no significant difference (*p* = 0.57) between male (*n* = 86, 41.5%) and female (*n* = 78, 39%) newborns. ABOs occurred in 119 (39.7%) of seronegative and 46 (42.2%) of seropositive women (*p* = 0.64). The difference in occurrence of SB (OR 0.81, *p* = 0.67), PTD (OR 1.01, *p* = 0.97) and SGA (OR 1.18, *p* = 0.63) was not significant according to serostatus (Table 3).

**Table 3:** Comparing different ARV exposure groups and adverse birth outcomes (ABOs)

Variables	Still-birth N (%) ^a^	OR	CI 95%	*P* value^b^	Preterm Delivery N (%)^c^	OR	CI 95%	*P* value^b^	Small for gest. age N (%)^d^	OR	CI 95%	*P* value^b^	Any ABO N (%)	OR	CI 95%	*P* value^b^
Drug exposure	25				112				44				158			
HIV negative	20 (6.6)	1			85 (28.2)	1			33 (11.3)	1			119 (39.7)	1		
ART pre-conception	3 (7.9)	1.21	0.34–4.28	0.73^e^	11 (28.9)	1.04	0.49–2.18	0.93	3 (8.3)	0.71	0.21–2.46	0.78^e^	13 (35.1)	0.82	0.4–1.68	0.59
Option B+	2 (3.4)	0.5	0.11–2.18	0.55^e^	16 (27.6)	0.97	0.52–1.81	0.92	8 (13.8)	1.3	0.55–2.88	0.59	26 (44.1)	1.2	0.68–2.11	0.53
ARV regimens	5				27				11				39			
ART pre-conception	3 (7.9)	1			11 (28.9)	1			3 (8.3)	1			13 (35.1)	1		
Option B+	2 (3.4)	0.41	0.07–2.57	0.38^e^	16 (27.6)	0.94	0.38–2.32	0.89	8 (13.8)	1.76	0.44–7.12	0.52^e^	26 (44.1)	1.46	0.62–3.4	0.39
Drug initiation^f^	5				27				11				39			
ARV exposure after GW 14	2 (4.3)	1			12 (26.7)	1			6 (13.3)	1			20 (43.5)	1		
ARV exposure before GW 14	3 (6.0)	1.4	0.22–8.81	1^e^	15 (30.0)	1.18	0.48–2.89	0.72	5 (10.4)	0.76	0.21–2.67	0.66	19 (38.8)	0,82	0.36–1.87	0.64
Any ARV intake ≥90 days prior delivery	5 (6.2)	1			21 (26.3)	1			8 (10.3)	1			8 (53.5)	1		
Any ARV intake <90 days prior delivery	0 (0)	0.84	0.76–0.92	1^e^	6 (40.0)	1.87	0.6–5.9	0.35^e^	3 (20.0)	0.46	0.11–1.97	0.38^e^	31 (38.8)	0.55	0.18–1.68	0.29
Exposure time	25				112				44				158			
HIV negative	20 (6.6)	1			85 (28.2)	1			33 (11.3)	1			119 (39.7)	1		
ARV exposure before GW 14	3 (6.0)	0.9	0.26–3.15	1^e^	15 (30.0)	1.09	0.57–2.1	0.8	5 (10.4)	0.91	0.34–2.47	0.86	19 (38.8)	0.96	0.52-1.79	0.91
Any ARV intake ≥90 days prior delivery	5 (5.2)	0.78	0.28–2.12	0.62	27 (28.4)	1.01	0.61–1.68	0.97	11 (11.8)	1.05	0.51–2.18	0.89	39 (41.1)	1.06	0.66–1.69	0.81
PMTCT intake prior delivery in days, Median (range)	135 (126–144)			0.86^g^	113,5 (14–278)			0.6^g^	125 (19–232)			0.73^g^	129 (14–278)			0.71^g^

^a^Total SB: 26; number excludes one woman who was on Option A at the time of delivery

^b^all data are bivariate, Pearson’s X^2^ asymptotic two- sided *p*- value, apart from where indicated differently

^c^Total PTD: 116; number excludes three women on Option A and one HIV positive woman without ARV treatment

^d^Total SGA: 47; number excludes two women on Option A and one woman with undocumented PMTCT regimen

^e^Bivariate Fisher’s Exact test two- sided *p*- value

^f^One woman on Option B+ excluded due to missing drug initiation date

^g^Mann–Whitney-U Test

There was also no difference in the occurrence of ABOs between the groups of exposure to ARVs. Among the 5 HIV-positive unexposed women, there was no ABO reported apart from one woman, who had anaemia and PTD at 37 GW. Compared to seronegative women, women on ARTpc did not have a significantly elevated risk for ABO occurrence (OR 0.82, CI 0.4–1.68, *p* = 0.59). The same was found for women on Option B+ (OR 1.2, CI 0.68–2.11, *p* = 0.53). When comparing the two groups of ARV exposure, ARTpc and Option B+, to each other, there was also no significance (OR 1.46, CI 0.62-3.4, *p* = 0.39) in ABO risk difference. No differing ABO risk could be reported for women who took ARV drugs longer or shorter than 90 days (OR 0.55, CI 0.18–1.68, *p* = 0.29) as well as among ARV-exposed and HIV-negative women during first trimester (OR 0.82, CI 0.36–1.87, *p* = 0.64).

### Stillbirth

In our cohort, 26/412 (6.3%) mothers delivered a stillborn infant. Women who had no formal education, were of poor SES, had hypertension, and who had anaemia were more likely to experience SB. SB occurred also more often to women who were referred to Virika Hospital from a distance of more than 90 min of travel time. In multivariate logistic regression, hypertension in pregnancy (AOR 18.03, CI 3.31–98.1, *p* = 0.001) and a travel distance to Virika Hospital of > 90 min (AOR 5.83, CI 2.21–15.42, *p* < 0.001) remained highly significant risk factors for SB (Table 4).

**Table 4:** Adverse birth outcome: Stillbirth (SB)

Variables	SB^a,b^	OR	CI 95%	P- value^c^	AOR	CI 95%	*P*- value
*N Total*	26						
Age	26						
<30 year	19 (6.2)	1					
≥30 year	7 (6.7)	1.08	0.44–2.65	0.86			
Education	26						
Primary and higher	21 (5.5)	1					
No formal education	5 (21.7)	4.78	1.62–14.12	*0.011* ^d^	1.12	0.22–5.67	0.89
Occupation	26						
Income generation	5 (5.0)	1					
No income generation	21 (7.2)	1.48	0.54–4.04	0.44			
Socioeconomic status (SES)	26						
Higher SES (≥1 assets)	15 (4.7)	1					
Lowest SES (0 assets)	11 (12.0)	2.75	1.22–6.22	*0.012*	1.89	0.69–5.17	0.21
Parity	26						
Primiparity	7 (5.3)	1					
Multiparity (≥2 deliveries)	19 (6.8)	1.29	0.53–3.15	0.58			
Travel distance	24						
<90 min	11 (3.7)	1					
≥90 min	13 (17.6)	5.6	2.4–13.1	*<0.001* ^d^	5.83	2.21–15.42	*<0.001*
Hypertension	26						
No hypertension	22 (5.5)	1					
Hypertension	4 (40)	11.52	3.03–43.81	*0.002*	18.03	3.31–98.1	*0.001*
Malaria in pregnancy	26						
No MIP detected peri-partum	24 (6.4)	1					
MIP detected peri-partum	2 (15.4)	2.64	0.55–12.61	0.22^d^			
MIP >3^rd^ trimester	3 (4.6)	1					
MIP ≤2^nd^ trimester or no MIP	23 (6.6)	1.47	0.43–5.04	0.78^d^			
Anaemia ≤11.5 mg/l	24						
No	12 (4.7)	1					
Yes	12 (10.3)	2.32	1.01–5.34	*0.042*	2.26	0.88–5.84	0.09

^a^All data N (%)

^b^Percentages refer to the number of participants with available data on respective variable

^c^Bivariate, Pearson’s X^2^ asymptotic two-sided *p*-value if not indicated otherwise. *P*-values in italics indicate statistically significant differences between the groups

^d^Bivariate, Fisher’s Exact test two- sided *p*- value

### Pre-term delivery

In 116 of 410 deliveries (missing gestational age in two cases), newborns were born pre-term (28.3%). PTD was more frequent in women of poor SES, lower education, primiparity, MIP in the last 2 weeks before delivery and anaemia (Table 5). Blood haemoglobin was on average 11.9 g/dl (5.9–15.9 g/dl, SD 1.87). Protective factors against PTD were tertiary education (OR 0.39, CI 0.16–0.96, *p* = 0.034), and higher SES (OR 0.47, CI 0.25–0.88, *p* = 0.008). After logistic regression, only anaemia (AOR 1.69, CI 1.01–2.84, *p* = 0.047) and MIP within the last 2 weeks before delivery (AOR 2.58, CI 1.03–6.46, *p* = 0.044) remained risk factors for PTD.

**Table 5:** Adverse birth outcome: Pre- term delivery (PTD)

Variables	PTD^a,b^	OR	CI 95%	P-value^c^	AOR	CI 95%	*P-* value
N total	116						
Age	116						
<30 year	84 (27.5)	1					
≥30 year	32 (30.8)	1.18	0.72–1.91	0.52			
Education	114						
Tertiary	6 (14.3)	1					
Secondary and less	108 (29.8)	2.55	1.04–6.23	*0.034*	1.67	0.63–4.4	0.3
Socioeconomic status (SES)	115						
Higher SES (≥1 assets)	79 (24.9)	1					
Lowest SES (0 assets)	36 (39.1)	1.94	1.19–3.16	*0.008*	1.48	0.72–3.06	0.29
ANC attendance	111						
Yes	106 (27.4)	1					
No	5 (62.5)	4.42	1.04–18.81	*0.043* ^d^	5.42	0.88–33.54	0.07
Parity	115						
Multiparity (≥2 deliveries)	68 (24.5)	1					
Primiparity	47 (35.9)	1.73	1.1–2.71	*0.017*	1.61	0.96–2.69	0.07
Malaria in pregnancy							
No MIP detected peri-partum	102 (27.5)/108	1					
MIP detected peri-partum	6 (46.2)	2.26	0.74–6.89	0.2			
MIP >2 weeks prior deliv. or no MIP	98 (26.1)/110	1					
≤2 weeks prior delivery	12 (48.0)	2.61	1.15–5.91	*0.018*	2.58	1.03–6.46	*0.044*
MIP >3^rd^ trimester	17 (26.2)/116	1					
MIP ≤2^nd^ trimester or no MIP	99 (28.7)	1.14	0.62–2.07	0.68			
Anaemia ≤11.5 mg/l	99						
No	60 (23.5)	1					
Yes	39 (33.6)	1.65	1.02–2.67	*0.04*	1.69	1.01–2.84	*0.047*

^a^All data N (%)

^b^Percentages refer to the number of participants with available data on respective variable

^c^Bivariate, Pearson’s X^2^ asymptotic two-sided *p*- value if not indicated otherwise. *P*-values in italics indicate statistically significant differences between the groups

^d^Bivariate, Fisher’s Exact test two- sided *p*- value

### Small for gestational age

Of 399 newborns with documented gestational duration and birth weight, 47 (11.8%) were SGA (Table 6). Comparing SGA occurrence among women with ARV intake >90 days to HIV-negative, ARV unexposed women, SGA risk was not different (Table 2). No significant correlations were found when assessing marital status, occupation, obstetric risk history, or haemoglobin level (Median 12.6 g/dl, SD 1.86, *p* = 0.89) for risk of SGA. In univariate analysis, women over the age of 30 years were more prone to deliver an SGA infant. If women experienced MIP, those with an SGA baby had the episode significantly later in pregnancy (Median 34.0 GW versus 28 GW, *p* = 0.02). After logistic regression, MIP in the third trimester was still a significant risk factor for SGA (Table 6).

**Table 6:** Adverse birth outcome: Small for Gestational Age (SGA)

Variables	SGA^a,b^	OR	CI 95%	P- value^c^	AOR	CI 95%	*P*- value
N total SGA	47						
Age	47						
≥30 year	7 (6.9)	1					
<30 year	40 (13.5)	2.11	0.92–4.88	*0.049*	2.09	0.9–4.85	0.085
Education	47						
No formal education	2 (9.1)	1					
Primary and higher	45 (12.1)	1.38	0.31–6.09	1.0^d^			
Socioeconomic status (SES)	47						
Higher SES (≥1 asset)	37 (12.0)	1					
Lowest SES (0 assets)	10 (11.1)	0.92	0.44–1.93	0.82			
Parity	47						
Grand multiparity ≥5 deliveries	2 (4.2)	1					
Parity ≤4 deliveries	45 (12.7)	3.21	0.75–13.72	0.1			
Malaria in pregnancy	47						
MIP detected peri-partum	1 (7.7)	1					
No MIP detected peri-partum	44 (12.2)	1.67	0.21–13.12	1.0^d^			
MIP ≤2^nd^ trimester or no MIP	34 (10.6)	1					
MIP >3^rd^ trimester	13 (20.3)	2.26	1.12–4.57	*0.02*	2.24	1.1–4.54	*0.026*
Anaemia ≤11.5 mg/l	46						
Yes	13 (11.8)	1					
No	31 (12.4)	1.06	0.53–2.11	0.88			

^a^All data N (%)

^b^Percentages refer to the number of participants with available data on respective variable

^c^Bivariate, Pearson’s X^2^ asymptotic two-sided *p*- value if not indicated otherwise. *P*-values in italics indicate statistically significant differences between the groups

^d^Bivariate, Fisher’s Exact test two- sided *p*- value

## Discussion

This cross-sectional, observational study assessed the occurrence of ABOs and their associations with maternal HIV status, ARV exposure and other influencing factors in a Western Ugandan health facility. PMTCT- Option B+ had recently been introduced here, and this is the first study to investigate possible associations between ABOs and Option B+ in this region.

In our cohort, the overall rate of ABOs was alarmingly high, and only few other studies, albeit also conducted in referral institutions, have reported similarly high rates [38–41].

Maternal ARV intake and maternal HIV infection have both been known to be potential risk factors for ABOs. While increased ABO risk is frequently reported for seropositive untreated women compared to HIV-negative women [42–44], we did not find a significant difference among seropositive women receiving ARVs and seronegative women. These results are in line with other publications [17, 39, 45–49]. The majority of HIV-positive women in our study had reported ARV exposure (ART or Option B+) exceeding 90 days, with presumably favourable effect on viral load and immunologic response by the time of delivery. Hence, our findings suggest a levelling-out effect of ARVs with respect to HIV infection as a risk factor for ABOs.

Among HIV-positive study participants, having started drug intake before conception (ARTpc) did not lead to a difference in ABO risk compared to having started only in pregnancy (Option B+). This is in accordance with findings from other studies, e.g. regarding PTD [22, 50] and SGA [17, 21, 48, 49]. For SB, our finding contradicts previous research, where higher risk for SB was reported among ARV-exposed women when intake started prior conception or early in pregnancy [11–13, 24, 40]. However, Cotter et al. [17] also did not observe risk differences for SB in relation to pre-conception ARV exposure. Among those initiating drug intake during pregnancy, length of Option B+ intake prior delivery did not play a significant role in our cohort.

In line with other studies, no difference was found for SGA risk among women taking ARV drugs and their seronegative counterparts. Several studies observed increased risks for PTD for women exposed to ARTpc containing PIs, probably caused by altered maternal progesterone levels [21, 24, 51, 52]. In our observational setting, apart from one woman, none took a regimen comprising PIs. The exposure to ARV combinations used in this study (N(t)RTIs and NNRTIS) did not have an increased risk for PTD and SB when compared to HIV-negative women, even if taken very early or throughout pregnancy. This confirms the result of other studies [17, 20, 34, 40, 53, 54]. At the same time, Marazzi et al. [34] could show a dramatic decrease of SB rate in women on ARV therapy compared to non-treated seropositive women. Hence, the potential toxicity of ARVs might be counterbalanced by the positive effect of the drugs on maternal HIV infection. ART programmes are being extensively scaled-up in most HIV-endemic countries, and therefore constantly increasing numbers of women in reproductive age will be enrolled in ART in the near future, while at the same time, more and more women will continue ART lifelong in the course of Option B+. Therefore it is an encouraging finding that ARV exposure does not seem to increase the risk for ABOs, even if taken for a long time.

In our study, ABO risk was negatively correlated to educational level and SES, a finding that has been well described in other settings [38, 55, 56]. We identified a number of clinical preconditions significantly linked with ABOs. A history of ABO was a predictor for repeated occurrence of the same event, which confirms previous research in other similar settings [38, 39, 43, 55, 57]. An increased risk of SB for women in our study with hypertension was accordingly reported in other studies [38, 56]. We also found that primigravid women were more prone to deliver a pre-term infant, supporting findings from Taha et al. [58]. Maternal anaemia was strongly correlated with SB and PTD in our study, also described by Watson-Jones et al. and Turner [55, 59]. Even though ANC at Virika Hospital routinely provides ferrous sulphate and folic acid, this does not seem to be enough to tackle the problem of anaemia. Laboratory haemoglobin testing during the antenatal period could help to filter out anaemic women and treat them according to the cause of anaemia. In general, our findings suggest that ANC surveillance to identify women with risk parameters needs to be strengthened, including assessment of the socio-economic situation and thorough history taking. Adequate blood pressure monitoring and early treatment of these women will contribute to lower the risk for ABOs. SB occurred to many women who were referred due to obstetrical complications. In line with other research [56], women with higher distance to the referral site and thus increased danger of foetal distress experienced SB more frequently. This implies that there is still need to improve the triage system for warning signs of ABO at the smaller health centres. Women with risk factors need to be referred already at ANC level, or early after their arrival for delivery. Also, better access, financially and transport-wise, needs to be reinforced to reach women in urgent need for adequate handling of risk pregnancies/deliveries. In our study setting, transport and cost coverage were provided to a part of the women, but these programs need to be expanded, and further research evaluating strategies for increasing accessibility is urgently required.

MIP turned out to be an important predictor of ABO occurrence. There was a strong correlation between MIP occurring later in pregnancy and SGA (3^rd^ trimester) as confirmed by Schmiegelow et al. [60], who found that sonographically assessed foetal growth was altered by MIP especially in the last trimester, even if women consequently received antimalarial treatment. Rijken et al. [61] found that a single or even asymptomatic MIP episode could cause foetal growth alteration. Landis et al. [62] observed high rates of SGA (29%) confirmed by ultrasound, equally linked with MIP. Similarly, we observed a higher occurrence of PTD for women with MIP within the last 2 weeks prior delivery, congruent with findings of other authors [63, 64]. MIP remains a crucial risk factor for ABO until late pregnancy [65, 66]. This implies a revision of strategies to prevent MIP for all women. Other research focussing on MIP in our study setting found that intermittent preventive treatment in pregnancy recommended for the region might no longer be adequate. High resistance of *Plasmodium falciparum* towards intermittent preventive treatment with sulfadoxine/pyrimethamine was detected, as reported in detail elsewhere [37]. Thus, immediate action is required to provide effective prophylaxis against MIP also with respect to ABO reduction in this setting.

Partly explained by the nature of our observational study setting, this research had some limitations. Since the number of ARV-unexposed seropositive women was very low, “HIV status” as an isolated risk factor as opposed to “ARV exposure” could not be assessed in this cohort. However, since the subgroup of HIV-infected while ARV-unexposed pregnant women will in general gradually disappear thanks to the universal scale-up of ART and Option B+, we believe that in the context of implementation research, it is not any longer a highly relevant comparison group. As another limitation, hospital staff often omitted peripartum HIV tests among presumably seronegative women, and we had to exclude numerous delivering women due to unconfirmed serostatus. Participants were also excluded due to missing newborn data. Infants who were stillborn or highly unstable after delivery were in some cases not assessed and weighed. However, the overall sample size is still considered large enough to provide valid findings with regard to our study question. We acknowledge that there might be other co- factors and conditions which were not assessed in our study, but which may also have played a role in influencing ABOs, like gestational diabetes or sexually transmitted infections, and these should be considered in future studies. Furthermore, we assessed gestational age with the Finnstroem score method. Finnstroem et al. describe a possibility of variation of up to 3 weeks in estimation of the GW through their method [30], so some misclassification is possible, especially in the transition zone of pre-term and term delivery. Lastly, regarding MIP as an important predictor for ABO, retrospective self-reported occurrence, timing and frequency of MIP need to be seen in the light of potential reporting bias. The same applies for the timing and intake of ARVs, antimalarial treatment and prophylaxis, as far as not noted on the ANC cards, as well as for obstetric and clinical history.

## Conclusions

While observed rates for ABOs in our study setting were overall considerably high, we did not find an adverse effect on birth outcomes for infants exposed to ARVs including both ARTpc and Option B+, independently from maternal intake duration. This is a promising result in the light of further roll- out of Option B+ and ART programmes in Subsaharan-African settings, especially considering the most recent “test and treat”- approach intending immediate treatment start for all HIV- infected individuals including pregnant women pursued by the WHO as well as by the Ugandan Ministry of Health after 2014 [8, 67]. Some other identified risk factors for ABOs, like hypertension, anaemia or delayed care caused by long travel distance might be avoided by improvement of ANC services, including better clinical screening and triage systems for timely referral. Also, effective preventive measures against MIP are urgently required to protect newborns from undesirable birth outcomes.

## Abbreviations

3TC: Lamivudine
ABO: Adverse birth outcome
ANC: Ante natal care
APGAR: Vigilance score for newborns developed by Victoria APGAR
APO: Adverse pregnancy outcome
ART: Anti-retroviral therapy
ARTpc: Highly active antiretroviral therapy, commenced prior conception of the pregnancy
ARV: Anti-retroviral
AZT: Zidovudine
EFV: Efavirenz
GW: Gestational week
HB: Haemoglobin
HIV: Human immunodeficiency virus (Type 1)
IPTp: Intermittent preventive treatment in pregnancy (against Malaria)
MIP: Malaria in pregnancy
N(t)RTI: Nucleoside (Nucleotide) reverse transcriptase inhibitor
NNRTI: Non- nucleoside reverse transcriptase inhibitor
Option A: ARV regimen to prevent vertical transmission of HIV, using AZT and Co- trimoxazole after GW 14 for PMTCT
OPTION B+: Regimen used to prevent vertical transmission of HIV, comprising TDF, 3TC and EFV
PMTCT: Prevention of mother-to-child-transmission of HIV
PTD: Preterm delivery
SB: Stillbirth
SES: Socioeconomic status
SGA: Small for gestational age
TDF: Tenofovir
WHO: World Health Organisation

## References

[1] De Cock KM, Fowler M, Mercier E (2000). Prevention of mother-to-child hiv transmission in resource-poor countries: Translating research into policy and practice. JAMA.

[2] Lallemant M, Chang S, Cohen R, Pecoul B (2011). Pediatric HIV — A Neglected Disease?. N Engl J Med.

[3] Tonwe-Gold B, Ekouevi DK, Viho I, Amani-Bosse C, Toure S, Coffie PA, Rouet F, Becquet R, Leroy V, El-Sadr WM (2007). Antiretroviral treatment and prevention of peripartum and postnatal HIV transmission in West Africa: evaluation of a two-tiered approach. PLoS Med.

[4] Steketee RW, Nahlen BL, Parise ME, Menendez C (2001). The burden of malaria in pregnancy in malaria-endemic areas. Am J Trop Med Hyg.

[5] Guyatt HL, Snow RW (2004). Impact of malaria during pregnancy on low birth weight in sub-Saharan Africa. Clin Microbiol Rev.

[6] Nkhoma ET, Kalilani-Phiri L, Mwapasa V, Rogerson SJ, Meshnick SR (2012). Effect of HIV infection and Plasmodium falciparum parasitemia on pregnancy outcomes in Malawi. Am J Trop Med Hyg.

[7] de Benoist B, McLEan E, Egli I, Cogswell M (2008). Worldwide prevalence of anaemia 1993–2005: WHO global database on anaemia. [Internet].

[8] WHO (2016). Consolidated guidelines on the use of antiretroviral drugs for treating and preventing HIV infection: recommendations for a public health approach [Internet].

[9] WHO (2012). Programmatic Update Guidelines Use of Antiretroviral Drugs for Treating Pregnant Women and Preventing HIV Infection in Infants. Executive Summary [Internet].

[10] Chen JY, Ribaudo HJ, Souda S, Parekh N, Ogwu A, Lockman S, Powis K, Dryden-Peterson S, Creek T, Jimbo W (2012). Highly active antiretroviral therapy and adverse birth outcomes among HIV-infected women in Botswana. J Infect Dis.

[11] Darak S, Darak T, Kulkarni S, Kulkarni V, Parchure R, Hutter I, Janssen F (2013). Effect of highly active antiretroviral treatment (HAART) during pregnancy on pregnancy outcomes: experiences from a PMTCT program in western India. AIDS Patient Care STDs.

[12] Thorne C, Rudin C, Newell ML, Kind C, Hug I, Gray L, Peckham CS (2000). Combination antiretroviral therapy and duration of pregnancy. AIDS (London, England).

[13] Boer K, Nellen JF, Patel D, Timmermans S, Tempelman C, Wibaut M, Sluman MA, van der Ende ME, Godfried MH (2007). The AmRo study: pregnancy outcome in HIV-1-infected women under effective highly active antiretroviral therapy and a policy of vaginal delivery. BJOG.

[14] Townsend CL, Byrne L, Cortina-Borja M, Thorne C, de Ruiter A, Lyall H, Taylor GP, Peckham CS, Tookey PA (2014). Earlier initiation of ART and further decline in mother-to-child HIV transmission rates, 2000–2011. AIDS (London, England).

[15] van der Merwe K, Hoffman R, Black V, Chersich M, Coovadia A, Rees H (2011). Birth outcomes in South African women receiving highly active antiretroviral therapy: a retrospective observational study. J Int AIDS Soc.

[16] Ekouevi DK, Coffie PA, Ouattara E, Moh R, Amani-Bosse C, Messou E, Sissoko M, Anglaret X, Eholie SP, Danel C (2011). Pregnancy outcomes in women exposed to efavirenz and nevirapine: an appraisal of the IeDEA West Africa and ANRS Databases, Abidjan, Cote d’Ivoire. J Acquir Immune Defic Syndr (1999).

[17] Cotter AM, Garcia AG, Duthely ML, Luke B, O’Sullivan MJ (2006). Is antiretroviral therapy during pregnancy associated with an increased risk of preterm delivery, low birth weight, or stillbirth?. J Infect Dis.

[18] McCormick MC (1985). The contribution of low birth weight to infant mortality and childhood morbidity. N Engl J Med.

[19] Liu L, Johnson HL, Cousens S, Perin J, Scott S, Lawn JE, Rudan I, Campbell H, Cibulskis R, Li M (2012). Global, regional, and national causes of child mortality: an updated systematic analysis for 2010 with time trends since 2000. Lancet.

[20] Grosch-Woerner I, Puch K, Maier RF, Niehues T, Notheis G, Patel D, Casteleyn S, Feiterna-Sperling C, Groeger S, Zaknun D (2008). Increased rate of prematurity associated with antenatal antiretroviral therapy in a German/Austrian cohort of HIV-1-infected women. HIV Med.

[21] Watts DH, Williams PL, Kacanek D, Griner R, Rich K, Hazra R, Mofenson LM, Mendez HA (2013). Combination antiretroviral use and preterm birth. J Infect Dis.

[22] Patel K, Shapiro DE, Brogly SB, Livingston EG, Stek AM, Bardeguez AD, Tuomala RE (2010). Prenatal protease inhibitor use and risk of preterm birth among HIV-infected women initiating antiretroviral drugs during pregnancy. J Infect Dis.

[23] Powis KM, Kitch D, Ogwu A, Hughes MD, Lockman S, Leidner J, van Widenfelt E, Moffat C, Moyo S, Makhema J (2011). Increased risk of preterm delivery among HIV-infected women randomized to protease versus nucleoside reverse transcriptase inhibitor-based HAART during pregnancy. J Infect Dis.

[24] Machado ES, Hofer CB, Costa TT, Nogueira SA, Oliveira RH, Abreu TF, Evangelista LA, Farias IF, Mercadante RT, Garcia MF (2009). Pregnancy outcome in women infected with HIV-1 receiving combination antiretroviral therapy before versus after conception. Sex Transm Infect.

[25] Patel D, Cortina-Borja M, Thorne C, Newell ML (2007). Time to undetectable viral load after highly active antiretroviral therapy initiation among HIV-infected pregnant women. Clin Infect Dis.

[26] UNAIDS. The Gap Report [Internet]. Geneva: UNAIDS. July 2014, updated September 2014 [cited 25 Dec 2015]. ISBN 978-92-9253-062-4 . Avaliable from: www.unaids.org/en/media/unaids/contentassets/documents/unaidspublication/2014/UNAIDS_Gap_report_en.pdf.

[27] UNICEF (2012). Countdown to Zero- Elimination of new infections among children until 2015 and keeping their mothers alive. Factsheet Uganda [Internet].

[28] Rubaihayo J, Akib S, Mughusu E, Abaasa A (2010). High HIV prevalence and associated factors in a remote community in the Rwenzori region of Western Uganda. Infect Dis Rep.

[29] Countdown to 2015 (2015). Uganda Accountability Profile [Internet].

[30] Finnstrom O (1977). Studies on maturity in newborn infants. IX. Further observations on the use of external characteristics in estimating gestational age. Acta Paediatr Scand.

[31] Schmiegelow C, Scheike T, Oesterholt M, Minja D, Pehrson C, Magistrado P, Lemnge M, Rasch V, Lusingu J, Theander TG (2012). Development of a fetal weight chart using serial trans-abdominal ultrasound in an East African population: a longitudinal observational study. PLoS One.

[32] Myer L (2011). Initiating antiretroviral therapy in pregnancy: the importance of timing. J Acquir Immune Defic Syndr (1999).

[33] Chibwesha CJ, Giganti MJ, Putta N, Chintu N, Mulindwa J, Dorton BJ, Chi BH, Stringer JSA, Stringer EM (2011). Optimal Time on HAART for Prevention of Mother-to-Child Transmission of HIV. J Acquir Immune Defic Syndr (1999).

[34] Marazzi MC, Palombi L, Nielsen-Saines K, Haswell J, Zimba I, Magid NA, Buonomo E, Scarcella P, Ceffa S, Paturzo G (2011). Extended antenatal use of triple antiretroviral therapy for prevention of mother-to-child transmission of HIV-1 correlates with favorable pregnancy outcomes. AIDS (London, England).

[35] Ekouevi DK, Coffie PA, Becquet R, Tonwe-Gold B, Horo A, Thiebaut R, Leroy V, Blanche S, Dabis F, Abrams EJ (2008). Antiretroviral therapy in pregnant women with advanced HIV disease and pregnancy outcomes in Abidjan, Cote d’Ivoire. AIDS (London, England).

[36] Suy A, Martinez E, Coll O, Lonca M, Palacio M, de Lazzari E, Larrousse M, Milinkovic A, Hernandez S, Blanco JL (2006). Increased risk of pre-eclampsia and fetal death in HIV-infected pregnant women receiving highly active antiretroviral therapy. AIDS (London, England).

[37] Braun V, Rempis E, Schnack A, Decker S, Rubaihayo J, Tumwesigye NM, Theuring S, Harms G, Busingye P, Mockenhaupt FP (2015). Lack of effect of intermittent preventive treatment for malaria in pregnancy and intense drug resistance in western Uganda. Malar J.

[38] Adane AA, Ayele TA, Ararsa LG, Bitew BD, Zeleke BM (2014). Adverse birth outcomes among deliveries at Gondar University Hospital, Northwest Ethiopia. BMC Pregnancy Childbirth.

[39] Lambert JS, Watts DH, Mofenson L, Stiehm ER, Harris DR, Bethel J, Whitehouse J, Jimenez E, Gandia J, Scott G (2000). Risk factors for preterm birth, low birth weight, and intrauterine growth retardation in infants born to HIV-infected pregnant women receiving zidovudine. Pediatric AIDS Clinical Trials Group 185 Team. AIDS (London, England).

[40] Zash R, Souda S, Chen JY, Binda K, Dryden-Peterson S, Lockman S, Mmalane M, Makhema J, Essex M et al. Reassuring Birth Outcomes with Tenofovir/Emtricitabine/Efavirenz used for Prevention of Mother to Child Transmission of HIV in Botswana. J Acquir Immune Defic Syndr. 2016;71(4):428–36. doi:10.1097/QAI.0000000000000847. 10.1097/QAI.0000000000000847 PMC4767604 26379069

[41] Ezechi OC, Gab-Okafor CV, Oladele DA, Kalejaiye OO, Oke BO, Ohwodo HO, Adu RA, Ekama SO, Musa Z, Onwujekwe DI (2013). Pregnancy, obstetric and neonatal outcomes in HIV positive Nigerian women. Afr J Reprod Health.

[42] Brocklehurst P, French R (1998). The association between maternal HIV infection and perinatal outcome: a systematic review of the literature and meta‐analysis. BJOG.

[43] Stringer EM, Vwalika B, Killam WP, Giganti MJ, Mbewe R, Chi BH, Chintu N, Rouse D, Goldenberg RL, Stringer JS (2011). Determinants of stillbirth in Zambia. Obstet Gynecol.

[44] Rollins NC, Coovadia HM, Bland RM, Coutsoudis A, Bennish ML, Patel D, Newell ML (2007). Pregnancy outcomes in HIV-infected and uninfected women in rural and urban South Africa. J Acquir Immune Defic Syndr (1999).

[45] Chi BH, Wang L, Read JS, Taha TE, Sinkala M, Brown ER, Valentine M, Martinson F, Goldenberg RL (2007). Predictors of stillbirth in sub-saharan Africa. Obstet Gynecol.

[46] Habib NA, Daltveit AK, Bergsjo P, Shao J, Oneko O, Lie RT (2008). Maternal HIV status and pregnancy outcomes in northeastern Tanzania: a registry-based study. BJOG.

[47] Haeri S, Shauer M, Dale M, Leslie J, Baker AM, Saddlemire S, Boggess K (2009). Obstetric and newborn infant outcomes in human immunodeficiency virus-infected women who receive highly active antiretroviral therapy. Am J Obstet Gynecol.

[48] Briand N, Mandelbrot L, Le Chenadec J, Tubiana R, Teglas JP, Faye A, Dollfus C, Rouzioux C, Blanche S, Warszawski J (2009). No relation between in-utero exposure to HAART and intrauterine growth retardation. AIDS (London, England).

[49] Marti C, Pena JM, Bates I, Madero R, de Jose I, Pallardo LF, Arribas JR, Gonzalez-Garcia J, Gonzalez A, Vazquez JJ (2007). Obstetric and perinatal complications in HIV-infected women. Analysis of a cohort of 167 pregnancies between 1997 and 2003. Acta Obstet Gynecol Scand.

[50] Tuomala RE, Shapiro DE, Mofenson LM, Bryson Y, Culnane M, Hughes MD, O’Sullivan MJ, Scott G, Stek AM, Wara D (2002). Antiretroviral Therapy during Pregnancy and the Risk of an Adverse Outcome. N Engl J Med.

[51] Szyld EGW, Eduardo M, Freimanis L, Gonin R, Cahn PE, Calvet GA, Duarte G, Melo VH, Read JS, for the NISDI Perinatal Study Group (2006). Maternal antiretroviral drugs during pregnancy and infant low birth weight and preterm birth. AIDS (London, England).

[52] Schulte J, Dominguez K, Sukalac T, Bohannon B, Fowler MG (2007). Declines in low birth weight and preterm birth among infants who were born to HIV-infected women during an era of increased use of maternal antiretroviral drugs: Pediatric Spectrum of HIV Disease, 1989–2004. Pediatrics.

[53] Martin F, Taylor GP (2007). Increased rates of preterm delivery are associated with the initiation of highly active antiretrovial therapy during pregnancy: a single-center cohort study. J Infect Dis.

[54] Kourtis AP, Schmid CH, Jamieson DJ, Lau J (2007). Use of antiretroviral therapy in pregnant HIV-infected women and the risk of premature delivery: a meta-analysis. AIDS (London, England).

[55] Watson-Jones D, Weiss HA, Changalucha JM, Todd J, Gumodoka B, Bulmer J, Balira R, Ross D, Mugeye K, Hayes R (2007). Adverse birth outcomes in United Republic of Tanzania: impact and prevention of maternal risk factors. Bull World Health Organ.

[56] Olusanya BO, Solanke OA (2009). Predictors of term stillbirths in an inner-city maternity hospital in Lagos, Nigeria. Acta Obstet Gynecol Scand.

[57] Folgosa E, Osman NB, Gonzalez C, Hagerstrand I, Bergstrom S, Ljungh A (1996). Syphilis seroprevalence among pregnant women and its role as a risk factor for stillbirth in Maputo, Mozambique. Genitourin Med.

[58] Taha TE, Dallabetta GA, Canner JK, Chiphangwi JD, Liomba G, Hoover DR, Miotti PG (1995). The effect of human immunodeficiency virus infection on birthweight, and infant and child mortality in urban Malawi. Int J Epidemiol.

[59] Turner AN, Tabbah S, Mwapasa V, Rogerson SJ, Meshnick SR, Ackerman W, Kwiek JJ (2013). Severity of maternal HIV-1 disease is associated with adverse birth outcomes in Malawian women: a cohort study. J Acquir Immune Defic Syndr (1999).

[60] Schmiegelow C, Minja D, Oesterholt M, Pehrson C, Suhrs HE, Bostrom S, Lemnge M, Magistrado P, Rasch V, Nielsen BB (2013). Malaria and fetal growth alterations in the 3(rd) trimester of pregnancy: a longitudinal ultrasound study. PLoS One.

[61] Rijken MJ, Papageorghiou AT, Thiptharakun S, Kiricharoen S, Dwell SL, Wiladphaingern J, Pimanpanarak M, Kennedy SH, Nosten F, McGready R (2012). Ultrasound evidence of early fetal growth restriction after maternal malaria infection. PLoS One.

[62] Landis SH, Lokomba V, Ananth CV, Atibu J, Ryder RW, Hartmann KE, Thorp JM, Tshefu A, Meshnick SR (2009). Impact of maternal malaria and under-nutrition on intrauterine growth restriction: a prospective ultrasound study in Democratic Republic of Congo. Epidemiol Infect.

[63] De Beaudrap P, Turyakira E, White LJ, Nabasumba C, Tumwebaze B, Muehlenbachs A, Guerin PJ, Boum Y, McGready R, Piola P (2013). Impact of malaria during pregnancy on pregnancy outcomes in a Ugandan prospective cohort with intensive malaria screening and prompt treatment. Malar J.

[64] Bardají A, Sigauque B, Sanz S, Maixenchs M, Ordi J, Aponte JJ, Mabunda S, Alonso PL, Menéndez C (2011). Impact of Malaria at the End of Pregnancy on Infant Mortality and Morbidity. J Infect Dis.

[65] Uneke CJ (2007). Impact of placental Plasmodium falciparum malaria on pregnancy and perinatal outcome in sub-Saharan Africa: I: introduction to placental malaria. Yale J Biol Med.

[66] Menendez C, Ordi J, Ismail MR, Ventura PJ, Aponte JJ, Kahigwa E, Font F, Alonso PL (2000). The impact of placental malaria on gestational age and birth weight. J Infect Dis.

[67] Uganda Ministry of Health (2013). Addendum to the national antiretroviral treatment guidelines [Internet].

